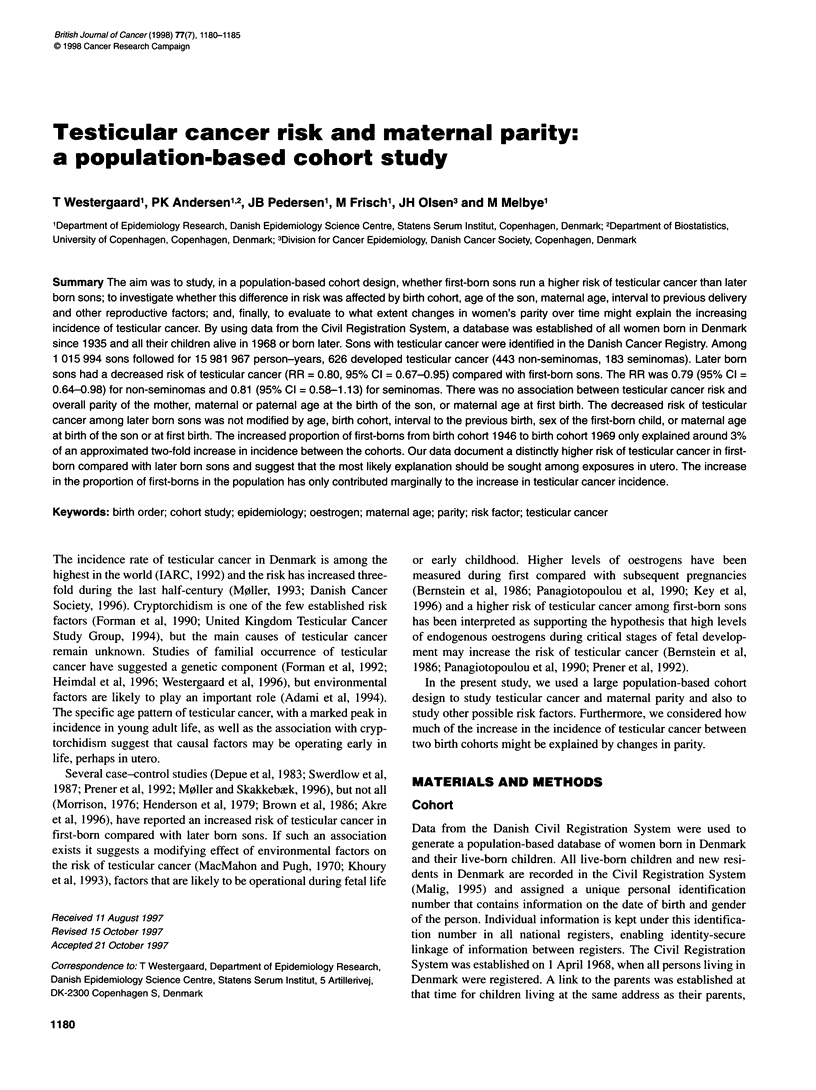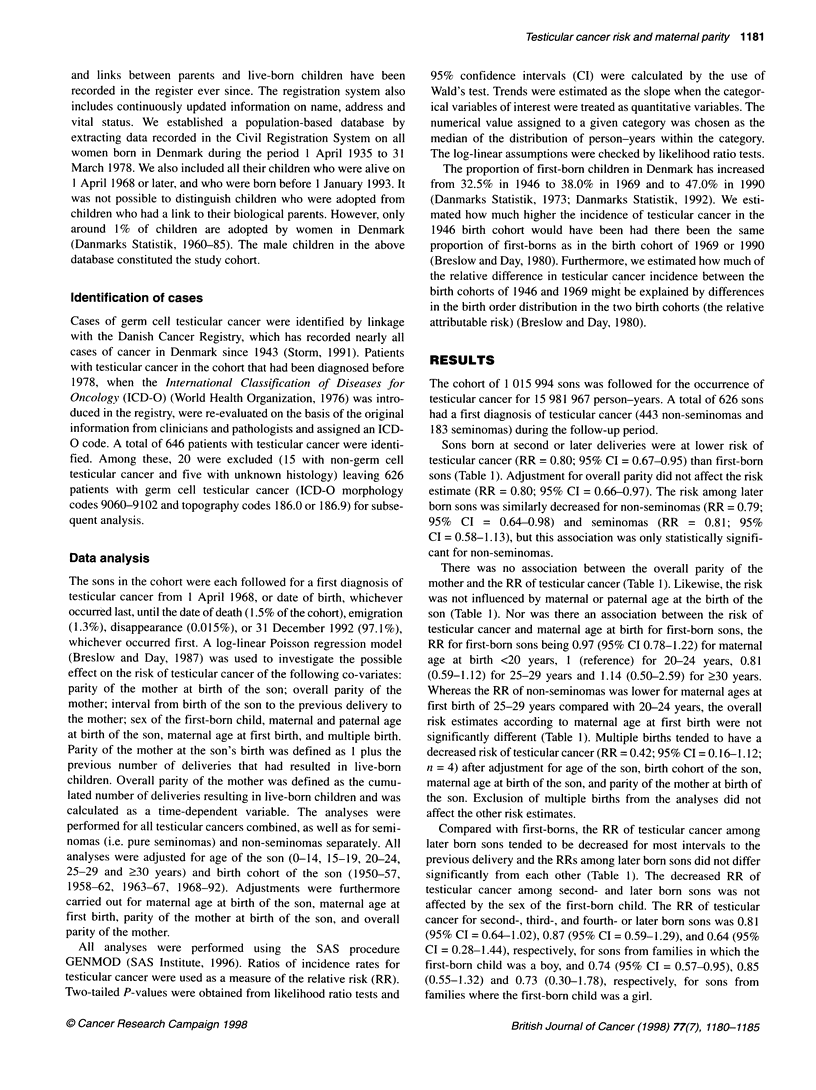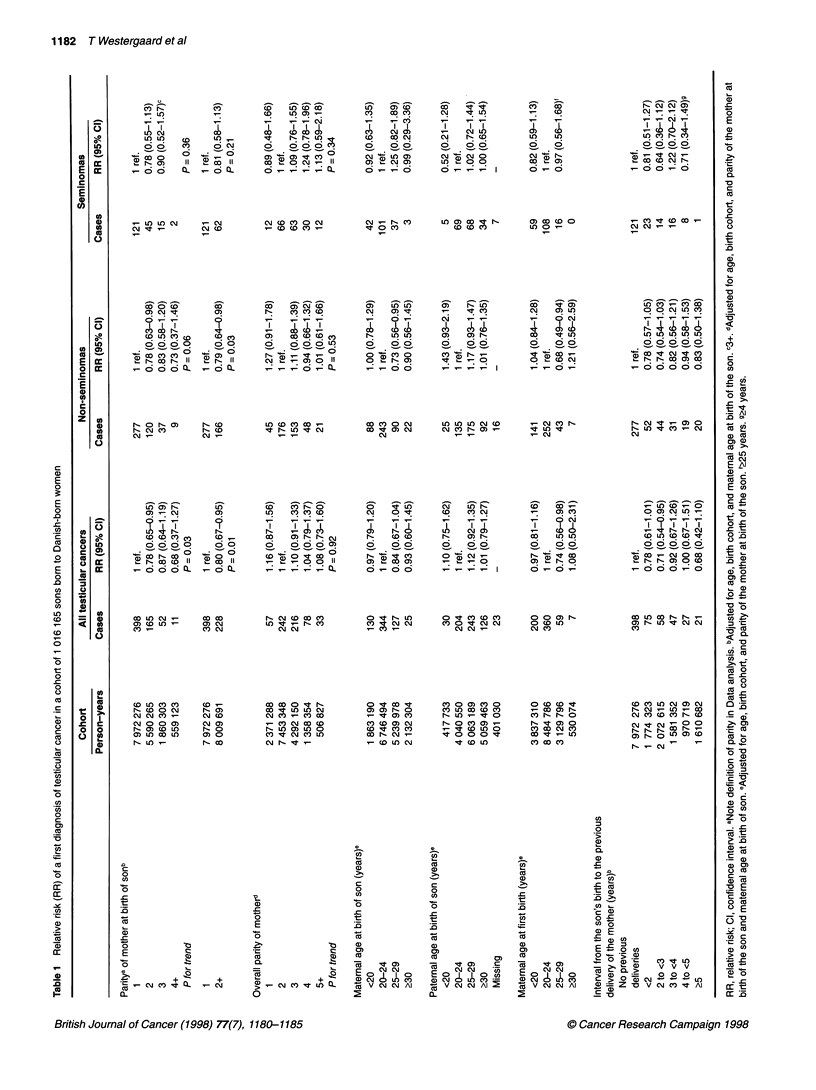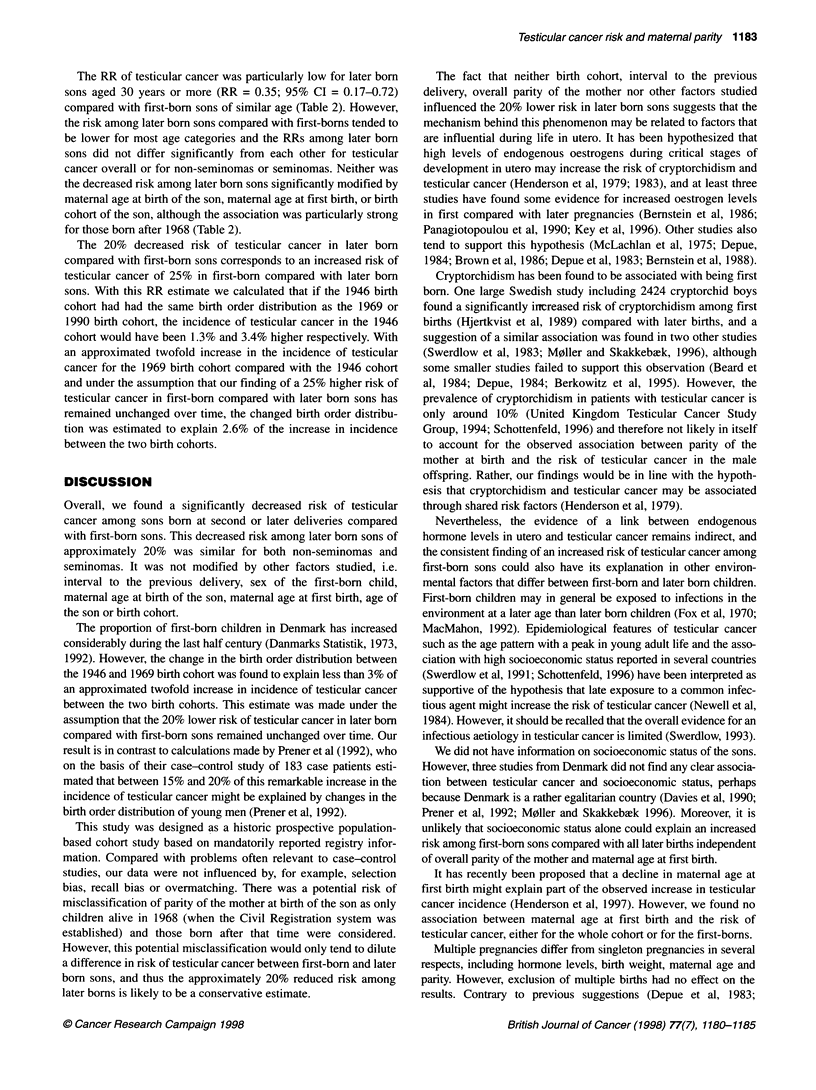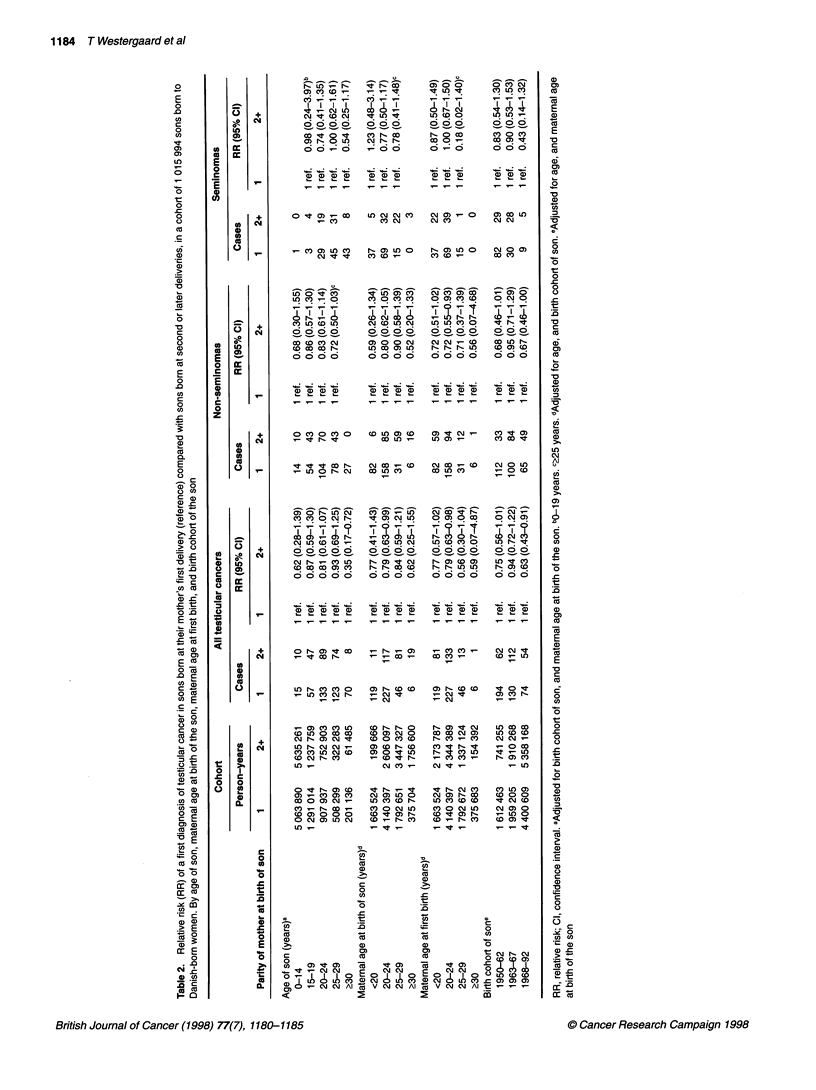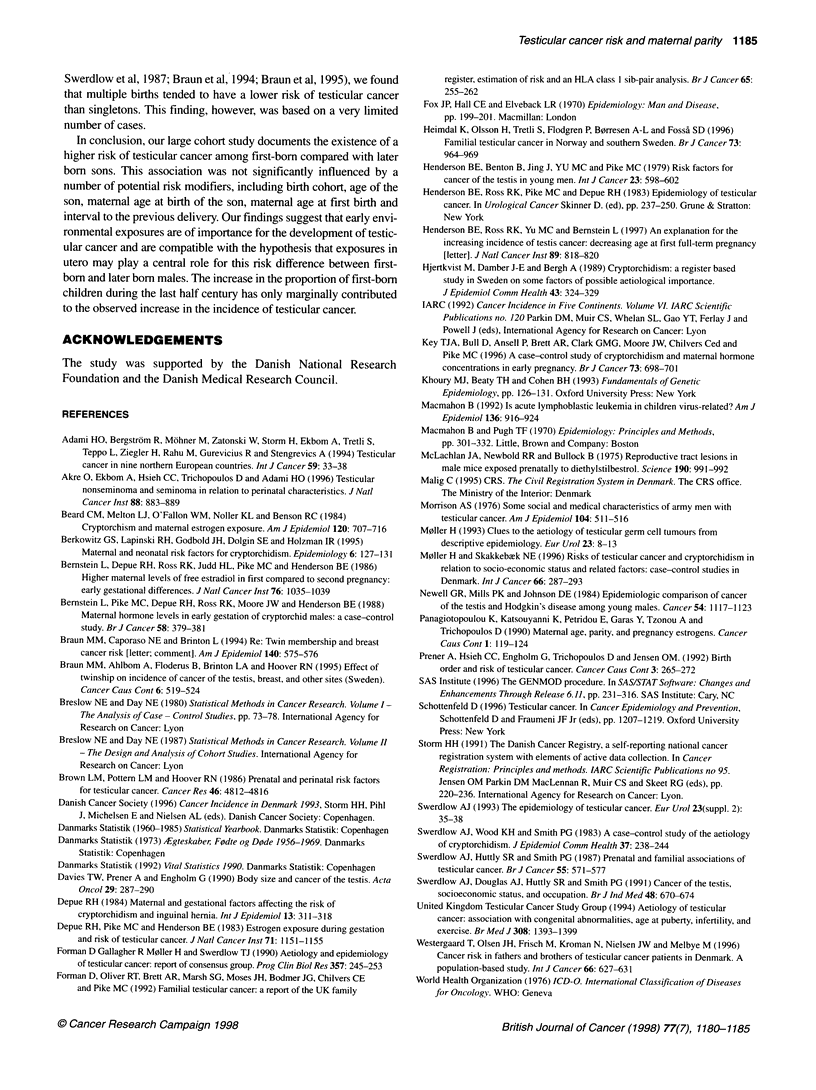# Testicular cancer risk and maternal parity: a population-based cohort study.

**DOI:** 10.1038/bjc.1998.196

**Published:** 1998-04

**Authors:** T. Westergaard, P. K. Andersen, J. B. Pedersen, M. Frisch, J. H. Olsen, M. Melbye

**Affiliations:** Department of Epidemiology Research, Danish Epidemiology Science Centre, Statens Serum Institut, Copenhagen.

## Abstract

The aim was to study, in a population-based cohort design, whether first-born sons run a higher risk of testicular cancer than later born sons; to investigate whether this difference in risk was affected by birth cohort, age of the son, maternal age, interval to previous delivery and other reproductive factors; and, finally, to evaluate to what extent changes in women's parity over time might explain the increasing incidence of testicular cancer. By using data from the Civil Registration System, a database was established of all women born in Denmark since 1935 and all their children alive in 1968 or born later. Sons with testicular cancer were identified in the Danish Cancer Registry. Among 1015994 sons followed for 15981 967 person-years, 626 developed testicular cancer (443 non-seminomas, 183 seminomas). Later born sons had a decreased risk of testicular cancer (RR = 0.80, 95% CI = 0.67-0.95) compared with first-born sons. The RR was 0.79 (95% CI = 0.64-0.98) for non-seminomas and 0.81 (95% CI = 0.58-1.13) for seminomas. There was no association between testicular cancer risk and overall parity of the mother, maternal or paternal age at the birth of the son, or maternal age at first birth. The decreased risk of testicular cancer among later born sons was not modified by age, birth cohort, interval to the previous birth, sex of the first-born child, or maternal age at birth of the son or at first birth. The increased proportion of first-borns from birth cohort 1946 to birth cohort 1969 only explained around 3% of an approximated two-fold increase in incidence between the cohorts. Our data document a distinctly higher risk of testicular cancer in first-born compared with later born sons and suggest that the most likely explanation should be sought among exposures in utero. The increase in the proportion of first-borns in the population has only contributed marginally to the increase in testicular cancer incidence.


*British Journal of Cancer* (1998) **77**(7), 1180–1185
© 1998 Cancer Research Campaign

### Testicular cancer risk and maternal parity: a population-based cohort study

**T Westergaard[1], PK Andersen[1,2], JB Pedersen[1], M Frisch[1], JH Olsen[3] and M Melbye[1]**

[1]Department of Epidemiology Research, Danish Epidemiology Science Centre, Statens Serum Institut, Copenhagen, Denmark; [2]Department of Biostatistics, University of Copenhagen, Copenhagen, Denmark; [3]Division for Cancer Epidemiology, Danish Cancer Society, Copenhagen, Denmark

**Summary** The aim was to study, in a population-based cohort design, whether first-born sons run a higher risk of testicular cancer than later born sons; to investigate whether this difference in risk was affected by birth cohort, age of the son, maternal age, interval to previous delivery and other reproductive factors; and, finally, to evaluate to what extent changes in women's parity over time might explain the increasing incidence of testicular cancer. By using data from the Civil Registration System, a database was established of all women born in Denmark since 1935 and all their children alive in 1968 or born later. Sons with testicular cancer were identified in the Danish Cancer Registry. Among 1 015 994 sons followed for 15 981 967 person–years, 626 developed testicular cancer (443 non-seminomas, 183 seminomas). Later born sons had a decreased risk of testicular cancer (RR = 0.80, 95% CI = 0.67–0.95) compared with first-born sons. The RR was 0.79 (95% CI = 0.64–0.98) for non-seminomas and 0.81 (95% CI = 0.58–1.13) for seminomas. There was no association between testicular cancer risk and overall parity of the mother, maternal or paternal age at the birth of the son, or maternal age at first birth. The decreased risk of testicular cancer among later born sons was not modified by age, birth cohort, interval to the previous birth, sex of the first-born child, or maternal age at birth of the son or at first birth. The increased proportion of first-borns from birth cohort 1946 to birth cohort 1969 only explained around 3% of an approximated two-fold increase in incidence between the cohorts. Our data document a distinctly higher risk of testicular cancer in first-born compared with later born sons and suggest that the most likely explanation should be sought among exposures in utero. The increase in the proportion of first-borns in the population has only contributed marginally to the increase in testicular cancer incidence.

**Keywords:** birth order; cohort study; epidemiology; oestrogen; maternal age; parity; risk factor; testicular cancer

The incidence rate of testicular cancer in Denmark is among the highest in the world (IARC, 1992) and the risk has increased three-fold during the last half-century (Møller, 1993; Danish Cancer Society, 1996). Cryptorchidism is one of the few established risk factors (Forman et al, 1990; United Kingdom Testicular Cancer Study Group, 1994), but the main causes of testicular cancer remain unknown. Studies of familial occurrence of testicular cancer have suggested a genetic component (Forman et al, 1992; Heimdal et al, 1996; Westergaard et al, 1996), but environmental factors are likely to play an important role (Adami et al, 1994). The specific age pattern of testicular cancer, with a marked peak in incidence in young adult life, as well as the association with cryptorchidism suggest that causal factors may be operating early in life, perhaps in utero.

Several case–control studies (Depue et al, 1983; Swerdlow et al, 1987; Prener et al, 1992; Møller and Skakkebæk, 1996), but not all (Morrison, 1976; Henderson et al, 1979; Brown et al, 1986; Akre et al, 1996), have reported an increased risk of testicular cancer in first-born compared with later born sons. If such an association exists it suggests a modifying effect of environmental factors on the risk of testicular cancer (MacMahon and Pugh, 1970; Khoury et al, 1993), factors that are likely to be operational during fetal life or early childhood. Higher levels of oestrogens have been measured during first compared with subsequent pregnancies (Bernstein et al, 1986; Panagiotopoulou et al, 1990; Key et al, 1996) and a higher risk of testicular cancer among first-born sons has been interpreted as supporting the hypothesis that high levels of endogenous oestrogens during critical stages of fetal development may increase the risk of testicular cancer (Bernstein et al, 1986; Panagiotopoulou et al, 1990; Prener et al, 1992).

In the present study, we used a large population-based cohort design to study testicular cancer and maternal parity and also to study other possible risk factors. Furthermore, we considered how much of the increase in the incidence of testicular cancer between two birth cohorts might be explained by changes in parity.

### MATERIALS AND METHODS

### Cohort

Data from the Danish Civil Registration System were used to generate a population-based database of women born in Denmark and their live-born children. All live-born children and new residents in Denmark are recorded in the Civil Registration System (Malig, 1995) and assigned a unique personal identification number that contains information on the date of birth and gender of the person. Individual information is kept under this identification number in all national registers, enabling identity-secure linkage of information between registers. The Civil Registration System was established on 1 April 1968, when all persons living in Denmark were registered. A link to the parents was established at that time for children living at the same address as their parents,

*Received 11 August 1997*
*Revised 15 October 1997*
*Accepted 21 October 1997*

*Correspondence to:* T Westergaard, Department of Epidemiology Research, Danish Epidemiology Science Centre, Statens Serum Institut, 5 Artillerivej, DK-2300 Copenhagen S, Denmark and links between parents and live-born children have been recorded in the register ever since. The registration system also includes continuously updated information on name, address and vital status. We established a population-based database by extracting data recorded in the Civil Registration System on all women born in Denmark during the period 1 April 1935 to 31 March 1978. We also included all their children who were alive on 1 April 1968 or later, and who were born before 1 January 1993. It was not possible to distinguish children who were adopted from children who had a link to their biological parents. However, only around 1% of children are adopted by women in Denmark (Danmarks Statistik, 1960–85). The male children in the above database constituted the study cohort.

### Identification of cases

Cases of germ cell testicular cancer were identified by linkage with the Danish Cancer Registry, which has recorded nearly all cases of cancer in Denmark since 1943 (Storm, 1991). Patients with testicular cancer in the cohort that had been diagnosed before 1978, when the *International Classification of Diseases for Oncology* (ICD-O) (World Health Organization, 1976) was introduced in the registry, were re-evaluated on the basis of the original information from clinicians and pathologists and assigned an ICD-O code. A total of 646 patients with testicular cancer were identified. Among these, 20 were excluded (15 with non-germ cell testicular cancer and five with unknown histology) leaving 626 patients with germ cell testicular cancer (ICD-O morphology codes 9060–9102 and topography codes 186.0 or 186.9) for subsequent analysis.

### Data analysis

The sons in the cohort were each followed for a first diagnosis of testicular cancer from 1 April 1968, or date of birth, whichever occurred last, until the date of death (1.5% of the cohort), emigration (1.3%), disappearance (0.015%), or 31 December 1992 (97.1%), whichever occurred first. A log-linear Poisson regression model (Breslow and Day, 1987) was used to investigate the possible effect on the risk of testicular cancer of the following co-variates: parity of the mother at birth of the son; overall parity of the mother; interval from birth of the son to the previous delivery to the mother; sex of the first-born child, maternal and paternal age at birth of the son, maternal age at first birth, and multiple birth. Parity of the mother at the son's birth was defined as 1 plus the previous number of deliveries that had resulted in live-born children. Overall parity of the mother was defined as the cumulated number of deliveries resulting in live-born children and was calculated as a time-dependent variable. The analyses were performed for all testicular cancers combined, as well as for seminomas (i.e. pure seminomas) and non-seminomas separately. All analyses were adjusted for age of the son (0–14, 15–19, 20–24, 25–29 and ≥30 years) and birth cohort of the son (1950–57, 1958–62, 1963–67, 1968–92). Adjustments were furthermore carried out for maternal age at birth of the son, maternal age at first birth, parity of the mother at birth of the son, and overall parity of the mother.

All analyses were performed using the SAS procedure GENMOD (SAS Institute, 1996). Ratios of incidence rates for testicular cancer were used as a measure of the relative risk (RR). Two-tailed $P$-values were obtained from likelihood ratio tests and 95% confidence intervals (CI) were calculated by the use of Wald's test. Trends were estimated as the slope when the categorical variables of interest were treated as quantitative variables. The numerical value assigned to a given category was chosen as the median of the distribution of person–years within the category. The log-linear assumptions were checked by likelihood ratio tests.

The proportion of first-born children in Denmark has increased from 32.5% in 1946 to 38.0% in 1969 and to 47.0% in 1990 (Danmarks Statistik, 1973; Danmarks Statistik, 1992). We estimated how much higher the incidence of testicular cancer in the 1946 birth cohort would have been had there been the same proportion of first-borns as in the birth cohort of 1969 or 1990 (Breslow and Day, 1980). Furthermore, we estimated how much of the relative difference in testicular cancer incidence between the birth cohorts of 1946 and 1969 might be explained by differences in the birth order distribution in the two birth cohorts (the relative attributable risk) (Breslow and Day, 1980).

### RESULTS

The cohort of 1 015 994 sons was followed for the occurrence of testicular cancer for 15 981 967 person–years. A total of 626 sons had a first diagnosis of testicular cancer (443 non-seminomas and 183 seminomas) during the follow-up period.

Sons born at second or later deliveries were at lower risk of testicular cancer (RR = 0.80; 95% CI = 0.67–0.95) than first-born sons (Table 1). Adjustment for overall parity did not affect the risk estimate (RR = 0.80; 95% CI = 0.66–0.97). The risk among later born sons was similarly decreased for non-seminomas (RR = 0.79; 95% CI = 0.64–0.98) and seminomas (RR = 0.81; 95% CI = 0.58–1.13), but this association was only statistically significant for non-seminomas.

There was no association between the overall parity of the mother and the RR of testicular cancer (Table 1). Likewise, the risk was not influenced by maternal or paternal age at the birth of the son (Table 1). Nor was there an association between the risk of testicular cancer and maternal age at birth for first-born sons, the RR for first-born sons being 0.97 (95% CI 0.78–1.22) for maternal age at birth <20 years, 1 (reference) for 20–24 years, 0.81 (0.59–1.12) for 25–29 years and 1.14 (0.50–2.59) for ≥30 years. Whereas the RR of non-seminomas was lower for maternal ages at first birth of 25–29 years compared with 20–24 years, the overall risk estimates according to maternal age at first birth were not significantly different (Table 1). Multiple births tended to have a decreased risk of testicular cancer (RR = 0.42; 95% CI = 0.16–1.12; $n$ = 4) after adjustment for age of the son, birth cohort of the son, maternal age at birth of the son, and parity of the mother at birth of the son. Exclusion of multiple births from the analyses did not affect the other risk estimates.

Compared with first-borns, the RR of testicular cancer among later born sons tended to be decreased for most intervals to the previous delivery and the RRs among later born sons did not differ significantly from each other (Table 1). The decreased RR of testicular cancer among second- and later born sons was not affected by the sex of the first-born child. The RR of testicular cancer for second-, third-, and fourth- or later born sons was 0.81 (95% CI = 0.64–1.02), 0.87 (95% CI = 0.59–1.29), and 0.64 (95% CI = 0.28–1.44), respectively, for sons from families in which the first-born child was a boy, and 0.74 (95% CI = 0.57–0.95), 0.85 (0.55–1.32) and 0.73 (0.30–1.78), respectively, for sons from families where the first-born child was a girl.

© Cancer Research Campaign 1998

© Cancer Research Campaign 1998

The RR of testicular cancer was particularly low for later born sons aged 30 years or more (RR = 0.35; 95% CI = 0.17–0.72) compared with first-born sons of similar age (Table 2). However, the risk among later born sons compared with first-borns tended to be lower for most age categories and the RRs among later born sons did not differ significantly from each other for testicular cancer overall or for non-seminomas or seminomas. Neither was the decreased risk among later born sons significantly modified by maternal age at birth of the son, maternal age at first birth, or birth cohort of the son, although the association was particularly strong for those born after 1968 (Table 2).

The 20% decreased risk of testicular cancer in later born compared with first-born sons corresponds to an increased risk of testicular cancer of 25% in first-born compared with later born sons. With this RR estimate we calculated that if the 1946 birth cohort had had the same birth order distribution as the 1969 or 1990 birth cohort, the incidence of testicular cancer in the 1946 cohort would have been 1.3% and 3.4% higher respectively. With an approximated twofold increase in the incidence of testicular cancer for the 1969 birth cohort compared with the 1946 cohort and under the assumption that our finding of a 25% higher risk of testicular cancer in first-born compared with later born sons has remained unchanged over time, the changed birth order distribution was estimated to explain 2.6% of the increase in incidence between the two birth cohorts.

### DISCUSSION

Overall, we found a significantly decreased risk of testicular cancer among sons born at second or later deliveries compared with first-born sons. This decreased risk among later born sons of approximately 20% was similar for both non-seminomas and seminomas. It was not modified by other factors studied, i.e. interval to the previous delivery, sex of the first-born child, maternal age at birth of the son, maternal age at first birth, age of the son or birth cohort.

The proportion of first-born children in Denmark has increased considerably during the last half century (Danmarks Statistik, 1973, 1992). However, the change in the birth order distribution between the 1946 and 1969 birth cohort was found to explain less than 3% of an approximated twofold increase in incidence of testicular cancer between the two birth cohorts. This estimate was made under the assumption that the 20% lower risk of testicular cancer in later born compared with first-born sons remained unchanged over time. Our result is in contrast to calculations made by Prener et al (1992), who on the basis of their case–control study of 183 case patients estimated that between 15% and 20% of this remarkable increase in the incidence of testicular cancer might be explained by changes in the birth order distribution of young men (Prener et al, 1992).

This study was designed as a historic prospective population-based cohort study based on mandatorily reported registry information. Compared with problems often relevant to case–control studies, our data were not influenced by, for example, selection bias, recall bias or overmatching. There was a potential risk of misclassification of parity of the mother at birth of the son as only children alive in 1968 (when the Civil Registration system was established) and those born after that time were considered. However, this potential misclassification would only tend to dilute a difference in risk of testicular cancer between first-born and later born sons, and thus the approximately 20% reduced risk among later borns is likely to be a conservative estimate.

The fact that neither birth cohort, interval to the previous delivery, overall parity of the mother nor other factors studied influenced the 20% lower risk in later born sons suggests that the mechanism behind this phenomenon may be related to factors that are influential during life in utero. It has been hypothesized that high levels of endogenous oestrogens during critical stages of development in utero may increase the risk of cryptorchidism and testicular cancer (Henderson et al, 1979; 1983), and at least three studies have found some evidence for increased oestrogen levels in first compared with later pregnancies (Bernstein et al, 1986; Panagiotopoulou et al, 1990; Key et al, 1996). Other studies also tend to support this hypothesis (McLachlan et al, 1975; Depue, 1984; Brown et al, 1986; Depue et al, 1983; Bernstein et al, 1988).

Cryptorchidism has been found to be associated with being first born. One large Swedish study including 2424 cryptorchid boys found a significantly increased risk of cryptorchidism among first births (Hjertkvist et al, 1989) compared with later births, and a suggestion of a similar association was found in two other studies (Swerdlow et al, 1983; Møller and Skakkebæk, 1996), although some smaller studies failed to support this observation (Beard et al, 1984; Depue, 1984; Berkowitz et al, 1995). However, the prevalence of cryptorchidism in patients with testicular cancer is only around 10% (United Kingdom Testicular Cancer Study Group, 1994; Schottenfeld, 1996) and therefore not likely in itself to account for the observed association between parity of the mother at birth and the risk of testicular cancer in the male offspring. Rather, our findings would be in line with the hypothesis that cryptorchidism and testicular cancer may be associated through shared risk factors (Henderson et al, 1979).

Nevertheless, the evidence of a link between endogenous hormone levels in utero and testicular cancer remains indirect, and the consistent finding of an increased risk of testicular cancer among first-born sons could also have its explanation in other environmental factors that differ between first-born and later born children. First-born children may in general be exposed to infections in the environment at a later age than later born children (Fox et al, 1970; MacMahon, 1992). Epidemiological features of testicular cancer such as the age pattern with a peak in young adult life and the association with high socioeconomic status reported in several countries (Swerdlow et al, 1991; Schottenfeld, 1996) have been interpreted as supportive of the hypothesis that late exposure to a common infectious agent might increase the risk of testicular cancer (Newell et al, 1984). However, it should be recalled that the overall evidence for an infectious aetiology in testicular cancer is limited (Swerdlow, 1993).

We did not have information on socioeconomic status of the sons. However, three studies from Denmark did not find any clear association between testicular cancer and socioeconomic status, perhaps because Denmark is a rather egalitarian country (Davies et al, 1990; Prener et al, 1992; Møller and Skakkebæk 1996). Moreover, it is unlikely that socioeconomic status alone could explain an increased risk among first-born sons compared with all later births independent of overall parity of the mother and maternal age at first birth.

It has recently been proposed that a decline in maternal age at first birth might explain part of the observed increase in testicular cancer incidence (Henderson et al, 1997). However, we found no association between maternal age at first birth and the risk of testicular cancer, either for the whole cohort or for the first-borns.

Multiple pregnancies differ from singleton pregnancies in several respects, including hormone levels, birth weight, maternal age and parity. However, exclusion of multiple births had no effect on the results. Contrary to previous suggestions (Depue et al, 1983;

© Cancer Research Campaign 1998

*© Cancer Research Campaign 1998*

Swerdlow et al, 1987; Braun et al, 1994; Braun et al, 1995), we found that multiple births tended to have a lower risk of testicular cancer than singletons. This finding, however, was based on a very limited number of cases.

In conclusion, our large cohort study documents the existence of a higher risk of testicular cancer among first-born compared with later born sons. This association was not significantly influenced by a number of potential risk modifiers, including birth cohort, age of the son, maternal age at birth of the son, maternal age at first birth and interval to the previous delivery. Our findings suggest that early environmental exposures are of importance for the development of testicular cancer and are compatible with the hypothesis that exposures in utero may play a central role for this risk difference between first-born and later born males. The increase in the proportion of first-born children during the last half century has only marginally contributed to the observed increase in the incidence of testicular cancer.

### ACKNOWLEDGEMENTS

The study was supported by the Danish National Research Foundation and the Danish Medical Research Council.

### REFERENCES

Adami HO, Bergström R, Möhner M, Zatonski W, Storm H, Ekbom A, Tretli S, Teppo L, Ziegler H, Rahu M, Gurevicius R and Stengrevics A (1994) Testicular cancer in nine northern European countries. *Int J Cancer* **59**: 33–38

Akre O, Ekbom A, Hsieh CC, Trichopoulos D and Adami HO (1996) Testicular nonseminoma and seminoma in relation to perinatal characteristics. *J Natl Cancer Inst* **88**: 883–889

Beard CM, Melton LJ, O'Fallon WM, Noller KL and Benson RC (1984) Cryptorchism and maternal estrogen exposure. *Am J Epidemiol* **120**: 707–716

Berkowitz GS, Lapinski RH, Godbold JH, Dolgin SE and Holzman IR (1995) Maternal and neonatal risk factors for cryptorchidism. *Epidemiology* **6**: 127–131

Bernstein L, Depue RH, Ross RK, Judd HL, Pike MC and Henderson BE (1986) Higher maternal levels of free estradiol in first compared to second pregnancy: early gestational differences. *J Natl Cancer Inst* **76**: 1035–1039

Bernstein L, Pike MC, Depue RH, Ross RK, Moore JW and Henderson BE (1988) Maternal hormone levels in early gestation of cryptorchid males: a case–control study. *Br J Cancer* **58**: 379–381

Braun MM, Caporaso NE and Brinton L (1994) Re: Twin membership and breast cancer risk [letter; comment]. *Am J Epidemiol* **140**: 575–576

Braun MM, Ahlbom A, Floderus B, Brinton LA and Hoover RN (1995) Effect of twinship on incidence of cancer of the testis, breast, and other sites (Sweden). *Cancer Caus Cont* **6**: 519–524

Breslow NE and Day NE (1980) *Statistical Methods in Cancer Research. Volume I – The Analysis of Case – Control Studies*, pp. 73–78. International Agency for Research on Cancer: Lyon

Breslow NE and Day NE (1987) *Statistical Methods in Cancer Research. Volume II – The Design and Analysis of Cohort Studies*. International Agency for Research on Cancer: Lyon

Brown LM, Pottern LM and Hoover RN (1986) Prenatal and perinatal risk factors for testicular cancer. *Cancer Res* **46**: 4812–4816

Danish Cancer Society (1996) *Cancer Incidence in Denmark 1993*, Storm HH, Pihl J, Michelsen E and Nielsen AL (eds). Danish Cancer Society: Copenhagen.

Danmarks Statistik (1960–1985) *Statistical Yearbook*. Danmarks Statistik: Copenhagen

Danmarks Statistik (1973) *Ægteskaber, Fødte og Døde 1956–1969*. Danmarks Statistik: Copenhagen

Danmarks Statistik (1992) *Vital Statistics 1990*. Danmarks Statistik: Copenhagen

Davies TW, Prener A and Engholm G (1990) Body size and cancer of the testis. *Acta Oncol* **29**: 287–290

Depue RH (1984) Maternal and gestational factors affecting the risk of cryptorchidism and inguinal hernia. *Int J Epidemiol* **13**: 311–318

Depue RH, Pike MC and Henderson BE (1983) Estrogen exposure during gestation and risk of testicular cancer. *J Natl Cancer Inst* **71**: 1151–1155

Forman D Gallagher R Møller H and Swerdlow TJ (1990) Aetiology and epidemiology of testicular cancer: report of consensus group. *Prog Clin Biol Res* **357**: 245–253

Forman D, Oliver RT, Brett AR, Marsh SG, Moses JH, Bodmer JG, Chilvers CE and Pike MC (1992) Familial testicular cancer: a report of the UK family register, estimation of risk and an HLA class 1 sib-pair analysis. *Br J Cancer* **65**: 255–262

Fox JP, Hall CE and Elveback LR (1970) *Epidemiology: Man and Disease*, pp. 199–201. Macmillan: London

Heimdal K, Olsson H, Tretli S, Flodgren P, Børresen A-L and Fosså SD (1996) Familial testicular cancer in Norway and southern Sweden. *Br J Cancer* **73**: 964–969

Henderson BE, Benton B, Jing J, YU MC and Pike MC (1979) Risk factors for cancer of the testis in young men. *Int J Cancer* **23**: 598–602

Henderson BE, Ross RK, Pike MC and Depue RH (1983) Epidemiology of testicular cancer. In *Urological Cancer* Skinner D. (ed), pp. 237–250. Grune & Stratton: New York

Henderson BE, Ross RK, Yu MC and Bernstein L (1997) An explanation for the increasing incidence of testis cancer: decreasing age at first full-term pregnancy [letter]. *J Natl Cancer Inst* **89**: 818–820

Hjertkvist M, Damber J-E and Bergh A (1989) Cryptorchidism: a register based study in Sweden on some factors of possible aetiological importance. *J Epidemiol Comm Health* **43**: 324–329

IARC (1992) *Cancer Incidence in Five Continents. Volume VI. IARC Scientific Publications no. 120* Parkin DM, Muir CS, Whelan SL, Gao YT, Ferlay J and Powell J (eds), International Agency for Research on Cancer: Lyon

Key TJA, Bull D, Ansell P, Brett AR, Clark GMG, Moore JW, Chilvers Ced and Pike MC (1996) A case–control study of cryptorchidism and maternal hormone concentrations in early pregnancy. *Br J Cancer* **73**: 698–701

Khoury MJ, Beaty TH and Cohen BH (1993) *Fundamentals of Genetic Epidemiology*, pp. 126–131. Oxford University Press: New York

Macmahon B (1992) Is acute lymphoblastic leukemia in children virus-related? *Am J Epidemiol* **136**: 916–924

Macmahon B and Pugh TF (1970) *Epidemiology: Principles and Methods*, pp. 301–332. Little, Brown and Company: Boston

McLachlan JA, Newbold RR and Bullock B (1975) Reproductive tract lesions in male mice exposed prenatally to diethylstilbestrol. *Science* **190**: 991–992

Malig C (1995) CRS. *The Civil Registration System in Denmark*. The CRS office. The Ministry of the Interior: Denmark

Morrison AS (1976) Some social and medical characteristics of army men with testicular cancer. *Am J Epidemiol* **104**: 511–516

Møller H (1993) Clues to the aetiology of testicular germ cell tumours from descriptive epidemiology. *Eur Urol* **23**: 8–13

Møller H and Skakkebæk NE (1996) Risks of testicular cancer and cryptorchidism in relation to socio-economic status and related factors: case–control studies in Denmark. *Int J Cancer* **66**: 287–293

Newell GR, Mills PK and Johnson DE (1984) Epidemiologic comparison of cancer of the testis and Hodgkin's disease among young males. *Cancer* **54**: 1117–1123

Panagiotopoulou K, Katsouyanni K, Petridou E, Garas Y, Tzonou A and Trichopoulos D (1990) Maternal age, parity, and pregnancy estrogens. *Cancer Caus Cont* **1**: 119–124

Prener A, Hsieh CC, Engholm G, Trichopoulos D and Jensen OM. (1992) Birth order and risk of testicular cancer. *Cancer Caus Cont* **3**: 265–272

SAS Institute (1996) The GENMOD procedure. In *SAS/STAT Software: Changes and Enhancements Through Release 6.11*, pp. 231–316. SAS Institute: Cary, NC

Schottenfeld D (1996) Testicular cancer. In *Cancer Epidemiology and Prevention*, Schottenfeld D and Fraumeni JF Jr (eds), pp. 1207–1219. Oxford University Press: New York

Storm HH (1991) The Danish Cancer Registry, a self-reporting national cancer registration system with elements of active data collection. In *Cancer Registration: Principles and methods. IARC Scientific Publications no 95*. Jensen OM Parkin DM MacLennan R, Muir CS and Skeet RG (eds), pp. 220–236. International Agency for Research on Cancer: Lyon.

Swerdlow AJ (1993) The epidemiology of testicular cancer. *Eur Urol* **23**(suppl. 2): 35–38

Swerdlow AJ, Wood KH and Smith PG (1983) A case–control study of the aetiology of cryptorchidism. *J Epidemiol Comm Health* **37**: 238–244

Swerdlow AJ, Huttly SR and Smith PG (1987) Prenatal and familial associations of testicular cancer. *Br J Cancer* **55**: 571–577

Swerdlow AJ, Douglas AJ, Huttly SR and Smith PG (1991) Cancer of the testis, socioeconomic status, and occupation. *Br J Ind Med* **48**: 670–674

United Kingdom Testicular Cancer Study Group (1994) Aetiology of testicular cancer: association with congenital abnormalities, age at puberty, infertility, and exercise. *Br Med J* **308**: 1393–1399

Westergaard T, Olsen JH, Frisch M, Kroman N, Nielsen JW and Melbye M (1996) Cancer risk in fathers and brothers of testicular cancer patients in Denmark. A population-based study. *Int J Cancer* **66**: 627–631

World Health Organization (1976) *ICD-O. International Classification of Diseases for Oncology*. WHO: Geneva

© Cancer Research Campaign 1998